# *CYP2C19* metabolizer phenotypes may affect the efficacy of statins on lowering small dense low-density lipoprotein cholesterol of patients with coronary artery disease

**DOI:** 10.3389/fcvm.2022.1016126

**Published:** 2022-12-19

**Authors:** Ruozhu Dai, Xiaoyu Zhao, Huilin Zhuo, Wei Wang, Yue Xu, Zixin Hu, Tiexu Zhang, Jiangman Zhao

**Affiliations:** ^1^Department of Cardiology, Quanzhou First Hospital Afliated to Fujian Medical University, Quanzhou, Fujian, China; ^2^Shanghai Biotecan Pharmaceuticals Co., Ltd., Shanghai Zhangjiang Institute of Medical Innovation, Shanghai, China; ^3^Artificial Intelligence Innovation and Incubation Institute, Fudan University, Shanghai, China; ^4^Fudan Zhangjiang Institute, Shanghai, China; ^5^Department of Cardiovascular Medicine, The First People's Hospital of Pingdingshan, Pingdingshan, Henan, China

**Keywords:** genetic polymorphisms, sdLDL-C, coronary artery disease, pharmacogenomics, *CYP2C19*

## Abstract

**Background:**

Dyslipidemia is a major cause of arteriosclerotic cardiovascular disease (ASCVD), and low-density lipoprotein cholesterol (LDL-C) is the profile to be reduced to prevent disease progression. Small dense low-density lipoprotein cholesterol (sdLDL-C) has been proven to be a more effective biomarker than LDL-C for ASCVD primary and secondary prevention. *CYP2C19* is an important drug metabolism gene. This study aimed to investigate the relationship between sdLDL-C and coronary artery disease (CAD) risk factors and explore the influence of *CYP2C19* metabolizer phenotypes on the sdLDL-C lowering efficacy of statins.

**Methods:**

This study recruited 182 patients with CAD and 200 non-CAD controls. Baseline laboratory indices of fasting blood were detected, including blood lipids, glucose, and creatinine. In addition, LDL-C subfractions were separated and quantified. Gene polymorphisms of *SLCO1B1* and *CYP2C19* were detected in patients with CAD. The LDL-C subfractions levels of patients with CAD were followed up after statin drug treatment.

**Results:**

Total cholesterol, LDL-C, LDLC-2, LDLC-3, LDLC-4, LDLC-5, LDLC-6, LDLC-7, and sdLDL-C levels of patients with CAD were significantly higher than those in non-CAD controls. Meanwhile, sdLDL-C (AUC = 0.838) and LDLC-4 (AUC = 0.835) performed outstandingly in distinguishing patients with CAD from controls. Based on *CYP2C19* metabolizer phenotypes, 113 patients with CAD were divided into the extensive metabolizer (EM, *n* = 49), intermediate metabolizer (IM, *n* = 52), and poor metabolizer (PM, *n* = 12) groups. The patients with IM and PM metabolizer phenotypes had better sdLDL-C lowering efficacy after taking statin drugs than patients with EM phenotype (*P* = 0.0268, FDR = 0.0536). The *SLCO1B1* genotype had no significant impact on the efficacy of statins (*P* = 0.1611, FDR = 0.1611).

**Conclusion:**

sdLDL-C and LDLC-4 outperformed other blood lipids such as LDL-C for CAD risk screening. *CYP2C19* metabolizer phenotypes had the potential to predict the efficacy of statins in lowering sdLDL-C.

## 1. Introduction

According to the 2019 Global Health Estimates by the World Health Organization, cardiovascular disease is the top cause of death worldwide, mainly involving ischemic heart disease and stroke (https://www.who.int/). In China, more than 10 million people suffer from CAD (1). Atherosclerosis is the main pathogenesis of many cardiovascular diseases, such as arteriosclerotic cardiovascular disease (ASCVD) (2). Hyperlipidemia is a well-studied risk factor for atherosclerosis (3). Dyslipidemia mainly refers to elevated low-density lipoprotein cholesterol (LDL-C) and triglycerides (TG) and reduced high-density lipoprotein cholesterol (HDL-C) (4). LDL-C is recognized as the most important factor to reveal the risk of ASCVD and is the main target to be controlled by lipid-lowering drugs for the primary and secondary prevention of ASCVD (5–8).

However, some studies stated that a normal level of LDL-C was observed in a significant percentage of patients with ASCVD (9). LDL-C was controlled to an ideal level, but the risk of cardiovascular events still exists (10, 11). LDL-C can be divided into Pattern A (large buoyant LDL-C, LDLC-1, and LDLC-2) and Pattern B (small dense LDL-C, LDLC-3, LDLC-4, LDLC-5, LDLC-6, and LDLC-7) based on its heterogeneous particles with various sizes, densities, and physicochemical properties (12). Increasing researchers reported that small dense LDL-C (sdLDL-C) was more atherogenic than large buoyant LDL-C (lbLDL-C) because of its easy oxidation, poor binding affinity with LDL receptors, a longer residual period in plasma, and greater penetration into the arterial wall (13–15). A Chinese large cohort study reported that sdLDL-C was independently related to carotid atherosclerosis progression (16). Further studies showed that sdLDL-C was associated with cardiovascular risk in patients with coronary artery disease (CAD) receiving statin treatment (13).

*SLCO1B1* c.521T>C (rs4149056) has been verified to be related to statin-induced myopathy risk, which is widely used to guide dose determination of statin drugs such as atorvastatin, pitavastatin, and simvastatin. Cytochrome P450 (CYP450) is a superfamily of genes encoding monooxygenases, which takes part in drug metabolism (17). The majority of hepatically cleared drugs are metabolized by CYP450 enzymes. *CYP2C19*, an important member of the CYP450 family, plays a role in the metabolism of commonly used clinical medicine, covering antiplatelet drugs, triazole antifungal agents, proton-pump inhibitors, antidepressants, and muscle relaxant analgesics (18). An individual carrying two no-function alleles of CYP2C19 (^*^2/^*^3) is designated as a *CYP2C19* poor metabolizer who has damaged pharmacodynamic responses to clopidogrel (19). Based on a comprehensive understanding of the role of variants of the *CYP2C19* genes on clopidogrel response, genetic screening may help decide the appropriate dose for patients (20). However, there is no study to evaluate the influence of *CYP2C19* metabolizer phenotypes on the efficacy of statin drugs, especially when sdLDL-C is used as a treatment target.

In this study, we recruited a total of 200 non-CAD controls and 182 newly diagnosed patients with CAD by coronary angiography from Quanzhou First Hospital Affiliated to Fujian Medical University and the First People's Hospital of Pingdingshan. The plasma lipids, especially LDL-C subfractions, were detected at the first visit of the patients. The genotypes of *SLCO1B1* and *CYP2C19* in patients with CAD were recognized. After the lipid-lowering treatment of statins, LDL-C subfractions were reviewed to assess the efficacy of statin treatment in individuals. We first assessed the risk factors for CAD development, especially comparing the levels of LDL-C subfractions between patients with CAD and controls. Then, we investigated the influence of *SLCO1B1* gene polymorphisms and *CYP2C19* metabolizer phenotypes on the sdLDL-C lowering efficacy of statin drugs in patients with CAD.

## 2. Materials and methods

### 2.1. Study population and sample collection

A total of 182 patients with CAD and 200 non-CAD controls were recruited from the Quanzhou First Hospital Affiliated to Fujian Medical University and the First People's Hospital of Pingdingshan from January 2018 to May 2020. Patients with CAD were diagnosed by coronary angiography. CAD is the condition where there is stenosis exceeding 50% in at least one branch of the coronary artery. The non-CAD controls were selected from a health examination population without a diagnosis or disease history of serious cardiovascular diseases such as ASCVD, stroke, carotid plaque, and so on. The participants who had received long-term lipid-lowering therapy or revascularization were excluded. The clinical information of participants was collected from their electronic medical record combing questionnaire, including age, gender, height, weight, drinking and smoking history, and disease history. Drinking history was defined as alcohol consumption (1) at least one time a week in the past 12 months or (2) of more than 30 g/day in the past 12 months. Any person who fulfilled this criterion but stopped drinking was also defined as a drinker. Smoking history was defined as follows: smokers include ex-smokers and current smokers, while non-smokers include those who had never smoked. Before any lipid-lowering treatment, overnight fasting blood was collected for laboratory tests of blood lipids and other indices such as fasting blood glucose (FBG) and serum creatinine (SCr). Among 182 patients with CAD, 113 patients received a *CYP2C19* genotyping test, and 172 patients received a *SLCO1B1* genotyping test.

**Figure 1:**
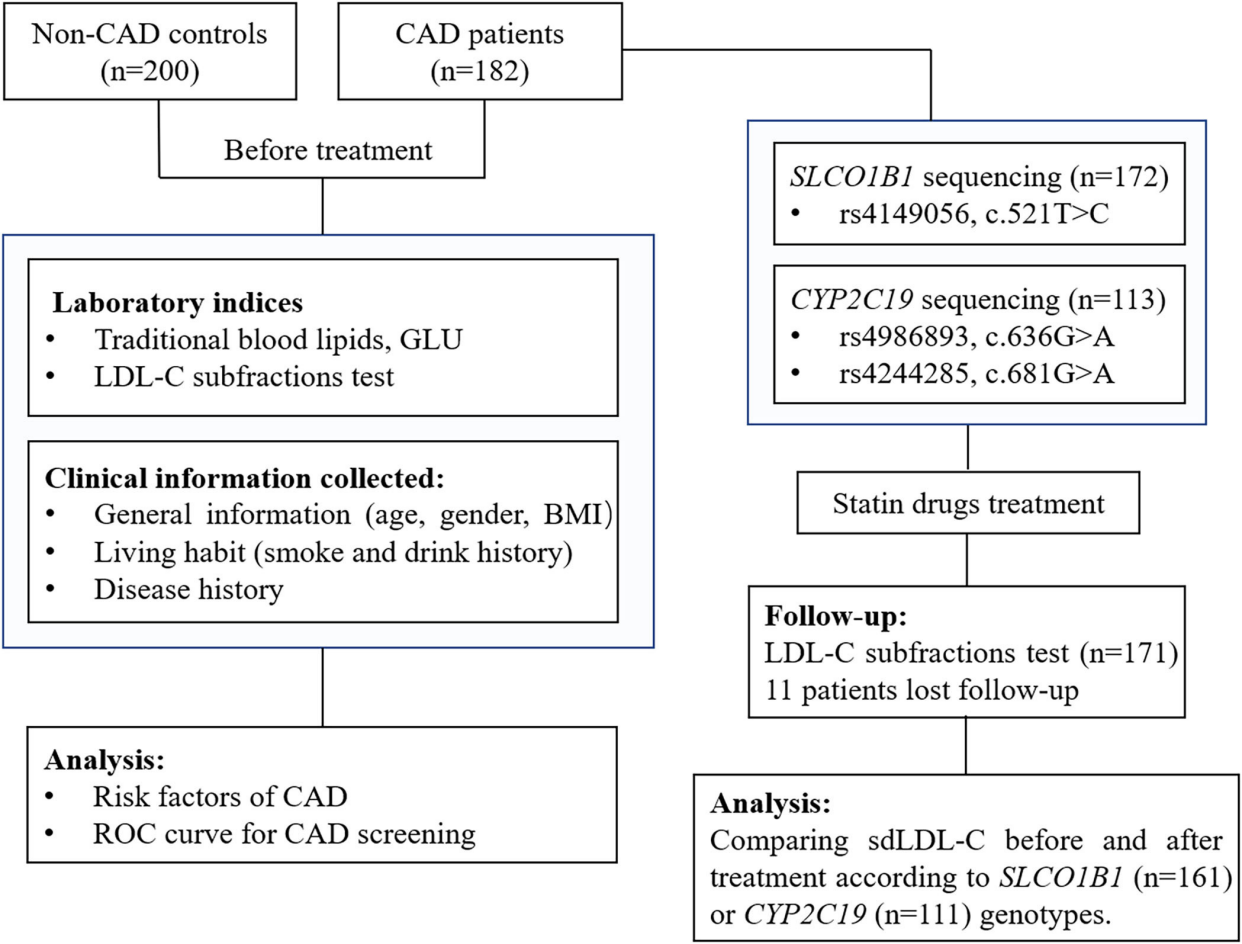
A flow diagram of this study. CAD, coronary artery disease; GLU, glucose; LDL-C, low-density lipoprotein cholesterol; BMI, body mass index; ROC curve, receiver operator characteristic curve.

After basic index testing and coronary angiography examination, patients with CAD were treated according to diagnosis results and clinical guidelines (21). The usage of lipid-lowering medications was determined based on clinical guidelines (4, 22, 23), mainly including atorvastatin, rosuvastatin, and pitavastatin. The LDL-C subfractions were reviewed in 171 patients after 3–6 months of lipid-lowering treatment. A total of eleven patients with CAD were lost to follow-up. The study design is shown in Figure 1.

All experimental protocols in this study were approved by the ethics committee of Quanzhou First Hospital Affiliated to Fujian Medical University and the First People's Hospital of Pingdingshan. All patients provided informed consent for this study. All methods in this study were carried out in accordance with the *Declaration of Helsinki*.

### 2.2. Laboratory index and LDL-C subtraction detection

Total cholesterol (TC), TG, HDL-C, LDL-C, SCr, and FBG were tested in the Department of Clinical Laboratory. For the primary prevention of ASCVD, the levels of TC, TG, and LDL-C that are equal or greater than 5.2 mmol/L, 1.7 mmol/L, and 3.4 mmol/L were defined as “marginal elevated”, and the levels that are equal or higher than 6.2 mmol/L, 2.3 mmol/L, and 4.1 mmol/L were defined as “elevated”, respectively, according to the 2016 Chinese guideline for the management of dyslipidemia in adult (22). LDL subfractions of plasma were separated and quantified by the LDL subfractions kit of Shanghai Biotecan Pharmaceuticals Co., Ltd. In brief, the plasma was mixed with Sudan Black B dye to stain the lipoproteins. Subsequently, the mixture was added to the top of precast polyacrylamide gel tubes. Then, samples were electrophoresed in electrophoresis apparatus for 70 min (3 mA/tub). Later, the densitometry of LDL subfractions was determined by Gel Scanner (Hunan Biotecan Medical Device Co., Ltd.). At last, the size-fractionated LDL-C was quantified using Gel Image Analysis Software of Gel Scanner (Hunan Biotecan Medical Device Co., Ltd.) according to the total TC value and the ratio of optical density value. LDL-C was then divided into 7 subfractions according to different sizes and densities, and sdLDL-C is defined as the sum of LDLC-3, LDLC-4, LDLC-5, LDLC-6, and LDLC-7.

### 2.3. *CYP2C19* and *SLOC1B1* genotyping

DNA was extracted from peripheral blood cells using the TIANamp Blood DNA Kit (No: DP348, TIANGEN, Beijing, China) following the protocol of the manufacturer. *SLCO1B1* (rs4149056, 521T>C), CYP2C19^*^2 (rs4244285, c.681G>A), and CYP2C19^*^3 (rs4986893, c.636G>A) were identified using the amplification-refractory mutation system (ARMS)-polymerase chain reaction (PCR) method.

### 2.4. Statistical analysis

Statistical analysis was performed using GraphPad Prism 6 (GraphPad Software, Inc., San Diego, CA, USA), IBM SPSS Statistics 22 (IBM, NY, USA), R project (R 4.0.2, R Core Team; https://www.R-Project.org), Stata 17.0 (StataCorp LLC, https://www.stata.com/), and PASS 2021 software (https://pass-software.com/). The categorical variables were presented by count number and percentage, and their distribution differences between groups were assessed with the chi-square test or Fisher's exact test. The continuous variables were presented by mean ± SD. Their differences were examined using a *t-*test when they followed a normal distribution; if not, then a non-parametric Mann–Whitney *U* test was performed. The interactive effect on blood lipids between the disease group and unbalanced clinical factors was analyzed using the general linear model of SPSS, and the adjusted *P*-value was calculated after covariate adjustment. The Spearman's rank coefficient of correlation among variables was performed using the R project. Univariate and multivariate logistic regression analyses were performed to investigate the independent risk factors of CAD risk. The receiver operator characteristic curve was used to evaluate the efficiency of biomarkers for distinguishing patients with CAD from controls. A P-value of < 0.05 indicated a statistically significant difference. For multiple testing corrections, the Benjamini–Hochberg method was used to control the false discovery rate (FDR). The PASS 2021 software was used to assess the power based on the given sample size.

## 3. Results

### 3.1. Clinical characteristics of patients with CAD and controls

**Figure 2:**
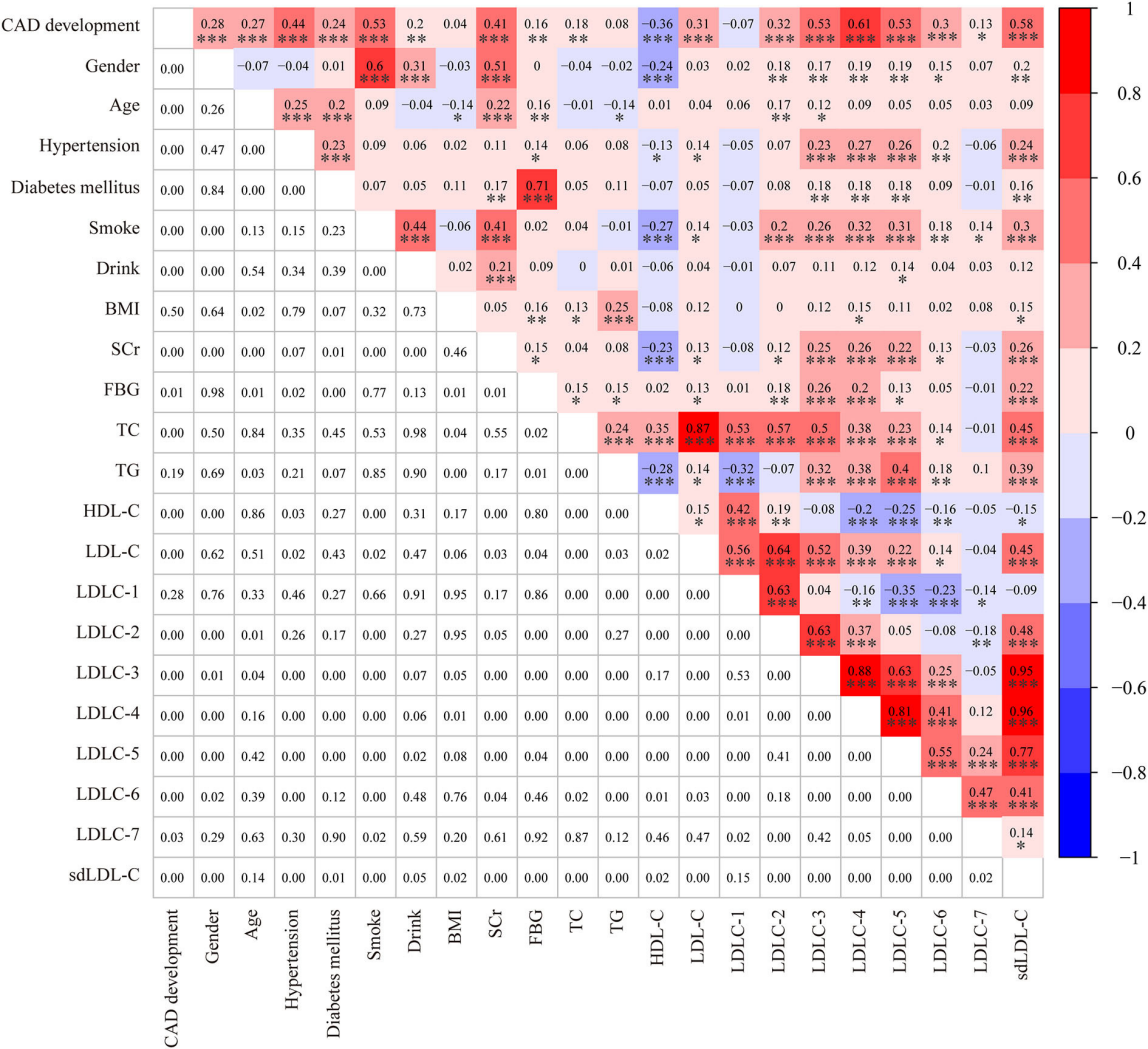
A heatmap showing the results of the Spearman correlation test among clinical characteristics and laboratory. The value in the grids of the upper triangle is the Spearman correlation coefficient (*r*), which is marked by colors. The value in grids of the lower triangle is the *P-*value of Spearman correlation. *P < 0.05, **P < 0.01, and ***P < 0.001.

Table 1 shows the clinical characteristics of 200 non-CAD controls and 182 patients with CAD. The majority of patients with CAD were men (77.5%), and the male ratio was significantly higher in patients with CAD than in controls (52.5%, *P* < 0.001). Meanwhile, there was a larger percentage of people with smoking (63.74 vs. 11.82%, *P* < 0.001) and drinking (25.27 vs. 10.91%, *P* = 0.003) history in patients with CAD than controls. The Spearman correlation analysis (Figure 2) showed male gender was strongly and positively correlated with smoking (*r* = 0.6, *P* < 0.001) and drinking history (*r* = 0.31, *P* < 0.001). These results indicated that men were the CAD high-risk population, mainly due to their unhealthy habits of drinking and smoking. In addition, a higher prevalence of hypertension (63.7 vs. 13.5%, *P* < 0.001) and diabetes mellitus (36.8 vs. 11.5%, *P* < 0.001) was observed in patients with CAD than that in controls. Figure 2 shows that hypertension was positively correlated with LDLC-3, LDLC-4, LDLC-5, LDLC-6, and sdLDL-C and that diabetes mellitus was also correlated with LDLC-3, LDLC-4, LDLC-5, and sdLDL-C.

**Table 1:** Clinical characteristics of CAD patients and non-CAD controls metabolizer status.

	**Controls (*n* = 200)**	**CAD patients (n = 182)**	**P value**
**Age**	**50.87 ± 13.66**	**64.14 ± 11.79**	**<0.001**
**Gender**			<0.001
Male	105 (52.5%)	141 (77.5%)	
Female	95 (47.5%)	41 (22.5%)	
**BMI**	24.02 ± 3.25	23.86 ± 3.50	0.787
**Smoke**			<0.001
Yes	13 (11.82%)	116 (63.74%)	
No	97 (88.18%)	66 (36.26%)	
Missing	90	0	
**Drink**			0.003
Yes	12 (10.91%)	46 (25.27%)	
No	98 (89.09)	136 (74.73%)	
Missing	90	0	
**Hypertension**			<0.001
Yes	27 (13.5%)	116 (63.7%)	
No	173 (86.5%)	66 (36.3%)	
**Diabetes mellitus**			<0.001
Yes	23 (11.5%)	67 (36.8%)	
No	177 (88.5%)	115 (63.2%)	

BMI, body mass index; CAD: coronary artery disease.

### 3.2. Comparison of blood lipids between patients with CAD and controls

**Figure 3:**
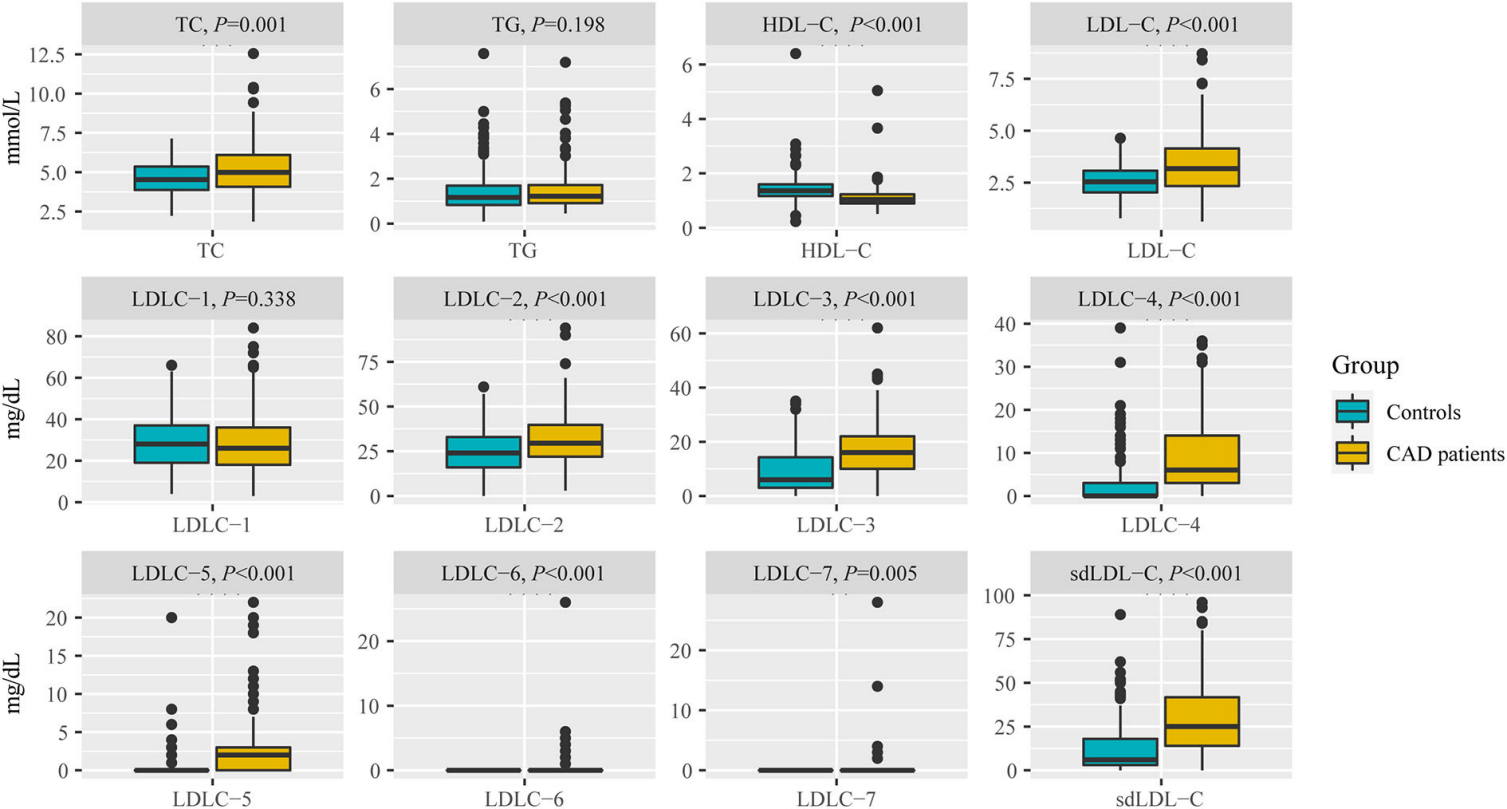
Comparison of blood lipids between non-CAD controls and patients with CAD, including TC, TG, HDL-C, LDL-C, LDLC-1 to LDLC-7, and sdLDL-C. TC, total cholesterol; TG, triglyceride; HDL-C, high-density lipoprotein cholesterol; LDL-C, low-density lipoprotein cholesterol; sdLDL-C, small dense low-density lipoprotein cholesterol.

Table 2 and Figure 3 indicate the level of HDL-C in patients with CAD was significantly lower than that in controls (1.12 ± 0.45 mmol/L vs. 1.42 ± 0.53 mmol/L, *P* < 0.001). The levels of TC (*P* < 0.001), LDL-C (*P* < 0.001), LDLC-2 (*P* < 0.001), LDLC-3 (*P* < 0.001), LDLC-4 (*P* < 0.001), LDLC-5 (*P* < 0.001), LDLC-6 (*P* < 0.001), LDLC-7 (*P* = 0.005), and sdLDL-C (*P* < 0.001) were significantly higher in patients with CAD than that in non-CAD controls. The interactive effect on blood lipids between the disease group and unbalanced clinical factors was analyzed using the general linear model of SPSS (Supplementary material 1). After adjusting covariates that had a significant interactive effect on blood lipid with the disease group, the adjusted *P*-value is shown in Supplementary material 1. After multiple testing, the FDR controlled by the Benjamini–Hochberg method is also shown in Supplementary material 1. There was a significant difference in the TG level (adjusted *P* = 0.013, FDR = 0.014) after adjusting for age and gender covariates and multiple testing corrections. For other lipids, the results after the adjustment of covariance and multiple testing correction were consistent with that before correction.

**Table 2:** Comparison of laboratory indices between non-CAD controls and CAD patients.

	**Controls**	**Patients**	**P value**
**TC (mmol/L)**	4.60 ± 1.046	5.19 ± 1.63	0.001
≥5.2 mmol/L	57/200 (28.50%)	78/171 (45.61%)	
≥6.2 mmol/L	14/200 (7.00%)	39/171 (22.81%)	
**TG (mmol/L)**	1.44 ± 0.97	1.52 ± 0.98	0.198
≥1.7 mmol/L	50/200 (25.00%)	45/171 (26.31%)	
≥2.3 mmol/L	24/200 (12.00%)	28/171 (16.37%)	
**HDL-C (mmol/L)**	1.42 ± 0.53	1.12 ± 0.45	<0.001
< 1.0 mmol/L	21/200 (10.50%)	66/170 (38.82%)	
**LDL-C (mmol/L)**	2.57 ± 0.77	3.31 ± 1.38	<0.001
≥3.4 mmol/L	27/200 (13.50%)	72/170 (42.35%)	
≥4.1 mmol/L	6/200 (3.00%)	46/170 (27.06%)	
**LDLC-1 (mg/dL)**	29.11 ± 12.93	28.83 ± 15.43	0.338
**LDLC-2 (mg/dL)**	25.29 ± 11.61	31.33 ± 14.16	<0.001
**LDLC-3 (mg/dL)**	9.53 ± 8.60	17.64 ± 10.19	<0.001
**LDLC-4 (mg/dL)**	2.57 ± 5.37	9.06 ± 8.18	<0.001
**LDLC-5 (mg/dL)**	0.32 ± 1.75	2.74 ± 4.02	<0.001
**LDLC-6 (mg/dL)**	0.00 ± 0.00	0.50 ± 2.14	<0.001
**LDLC-7 (mg/dL)**	0.00 ± 0.00	0.31 ± 2.36	0.005
**sdLDL-C (mg/dL)**	12.43 ± 13.90	30.24 ± 20.80	<0.001
**FBG (mmol/L)**	5.79 ± 1.96	6.64 ± 2.70	0.001
**SCr (umol/L)**	65.56 ± 14.50	80.80 ± 27.35	<0.001

TC, total cholesterol; TG, triglyceride; HDL-C, high-density lipoprotein cholesterol; LDL-C, low-density lipoprotein cholesterol; sdLDL-C, small and dense low-density lipoprotein cholesterol; FBG, fasting blood glucose; SCr, serum creatinine.

The marginally elevated ratios of TC, TG, and LDL-C in controls and patients with CAD were 28.50 vs. 45.61%, 25.00 vs. 26.31%, and 13.50 vs. 42.35%, respectively (Table 2). The elevated ratios of TC, TG, and LDL-C in controls and patients with CAD were 7.00 vs. 22.81%, 12.00 vs. 16.37%, and 3.00 vs. 27.06%, respectively (Table 2). These results indicated that a substantial portion of patients with CAD risk will be missed for further examination by screening traditional blood lipids such as TC, TG, and LDL-C.

### 3.3. Risk factors of CAD development by logistic regression analysis

A univariate logistic regression analysis was used to identify the risk factors of CAD development (Table 3). The results indicated that older age (OR = 1.084, *P* < 0.001), male gender (OR = 3.111, *P* < 0.001), hypertension (OR = 11.262, *P* < 0.001), diabetes (OR = 4.484, *P* < 0.001), smoking (OR = 13.114, *P* < 0.001), and drinking (OR = 2.762, *P* = 0.004) history were all the clinical risk factors of CAD development. For blood lipids, the elevated TC (OR = 1.402, *P* < 0.001), LDL-C (OR = 1.952, *P* < 0.001), LDLC-2 (OR = 1.038, *P* < 0.001), LDLC-3 (OR = 1.100, *P* < 0.001), LDLC-4 (OR = 1.182, *P* < 0.001), LDLC-5 (OR = 1.804, *P* < 0.001), and sdLDL-C (OR = 1.067, *P* < 0.001) were risk factors of CAD development. Elevated HDL-C was a protective factor of CAD development (OR = 0.121, *P* < 0.001). Multivariate logistic regression analysis was performed to further investigate the independent correlation between blood lipids and CAD risk by adjusting clinical risk factors, including age, gender, hypertension, diabetes, smoking, and drinking history. The results showed that TC (OR = 1.565, *P* < 0.001), LDL-C (OR = 2.142, *P* < 0.001), sdLDL-C (OR = 1.085, *P* < 0.001), LDLC-2 (OR = 1.056, *P* < 0.001), LDLC-3 (OR = 1.132, *P* < 0.001), LDLC-4 (OR = 1.214, *P* < 0.001), and LDLC-5 (OR = 1.843, *P* < 0.001) were all independent risk factors of CAD risk (Table 4).

**Table 3:** Risk factors of CAD by logistic regression analysis.

**Variables**	***P*** **value**	**Wald**	**OR (95% CI)**
**Age**	<0.001	66.051	1.084 (1.063-1.105)
**Male gender**	<0.001	25.003	3.111 (1.994-4.855)
**Hypertension**	<0.001	88.049	11.262 (6.791-18.674)
**Diabetes**	<0.001	30.945	4.484 (2.643-7.607)
**Smoke**	<0.001	59.672	13.114 (6.826-25.196)
**Drink**	0.004	8.418	2.762 (1.392-5.487)
**BMI**	0.917	0.011	0.997(0.935-1.063)
**TC**	<0.001	15.747	1.402 (1.187-1.657)
**TG**	0.446	0.581	1.085 (0.879-1.339)
**HDL-C**	<0.001	34.154	0.121 (0.060-0.246)
**LDL-C**	<0.001	33.541	1.952 (1.557-2.449)
**SdLDL-C**	<0.001	60.322	1.067 (1.050-1.085)
**LDLC-1**	0.847	0.037	0.999 (0.985-1.013)
**LDLC-2**	<0.001	18.627	1.038 (1.021-1.056)
**LDLC-3**	<0.001	51.088	1.100 (1.072-1.129)
**LDLC-4**	<0.001	51.792	1.182 (1.130-1.238)
**LDLC-5**	<0.001	38.489	1.804 (1.497-2.174)

**Table 4:** Multivariate logistic regression analysis of the independent correlation between blood lipids and CAD risk, by adjusting clinical risk factors.

**Variables**	***P*** **value**	**Wald**	**OR (95% CI)**
TC model	<0.001	12.380	1.565 (1.219–2.008)
HDL-C model	0.008	6.981	0.384 (0.189–0.781)
LDL-C model	<0.001	19.345	2.142 (1.526–3.009)
SdLDL-C model	<0.001	31.161	1.085 (1.054–1.116)
LDLC-2 model	<0.001	15.226	1.056 (1.027–1.085)
LDLC-3 model	<0.001	29.735	1.132 (1.083–1.184)
LDLC-4 model	<0.001	26.011	1.214 (1.127–1.308)
LDLC-5 model	<0.001	16.611	1.843 (1.374–2.474)

Every model additionally adjusted for age, gender, hypertension, diabetes, smoke, and drink.

### 3.4. The performance of blood lipids to distinguish patients with CAD from controls

**Figure 4:**
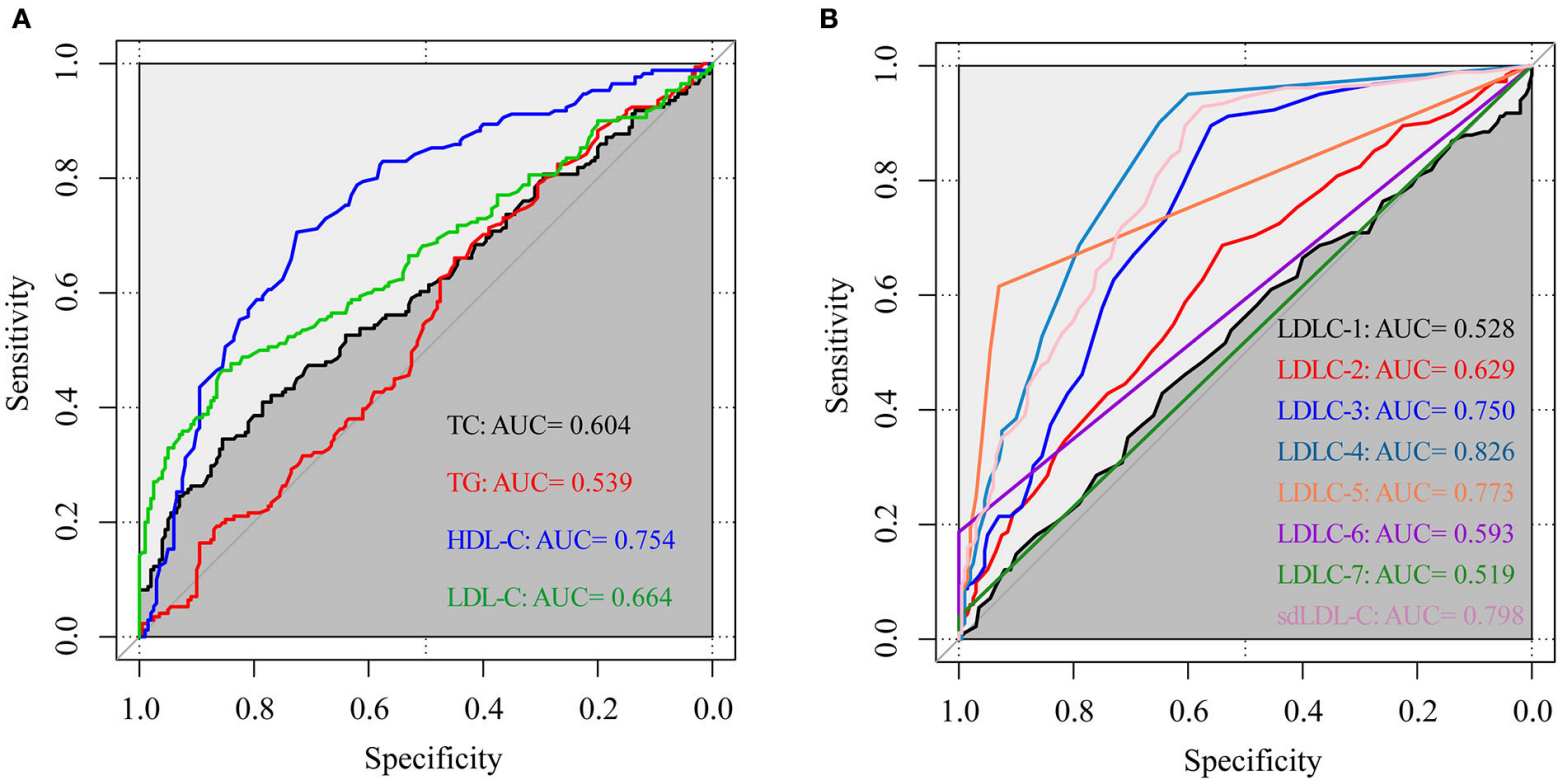
The ROC curve of blood lipids to distinguish patients with CAD from non-CAD controls. **(A)** TC, TG, HDL-C, and LDL-C; **(B)** LDL-C subfractions (LDLC-1 to LDLC-7) and sdLDL-C. ROC curve, receiver operator characteristic curve; AUC, area under the curve; TC, total cholesterol; TG, triglyceride; HDL-C, high-density lipoprotein cholesterol; LDL-C, low-density lipoprotein cholesterol; sdLDL-C, small dense low-density lipoprotein cholesterol.

Figure 4 shows the results of the ROC curve for blood lipids to distinguish patients with CAD from non-CAD controls. The AUC values of TC, TG, HDL-C, and LDL-C were, respectively, 0.604, 0.539, 0.754, and 0.664. The AUC values of LDLC-1, LDLC-2, LDLC-3, LDLC-4, LDLC-5, LDLC-6, LDLC-7, and sdLDL-C were, respectively, 0.528, 0.629, 0.750, 0.826, 0.773, 0.593, 0.519, and 0.798. These results indicated that LDLC-4 had the best capability (AUC = 0.826) for CAD screening in the health examination population. Total sdLDL-C took the second place for CAD screening (AUC = 0.798).

**Figure 5:**
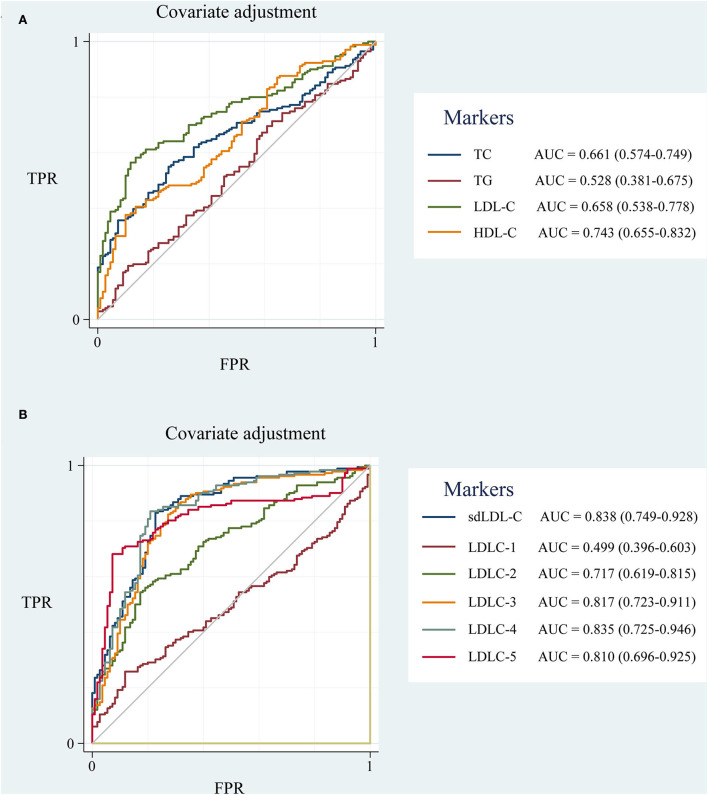
The ROC curve of blood lipids adjusted for covariates of age, gender, hypertension and diabetes disease history, drinking and smoking history. **(A)** TC, TG, HDL-C, and LDL-C; **(B)** LDL-C subfractions (LDLC-1 to LDLC-7) and sdLDL-C. ROC curve, receiver operator characteristic curve; AUC, area under the curve; TC, total cholesterol; TG, triglyceride; HDL-C, high-density lipoprotein cholesterol; LDL-C, low-density lipoprotein cholesterol; sdLDL-C, small dense low-density lipoprotein cholesterol; TPR, true positive rate; FPR, false positive rate.

To eliminate the influence of unbalanced clinical factors, the ROC curves were adjusted by covariates of age, gender, hypertension and diabetes disease history, and drinking and smoking history using Stata 17.0 software (Figure 5). sdLDL-C and LDLC-4 still showed a comparable outstanding AUC value of 0.838 and 0.835, respectively.

### 3.5. Influence of *CYP2C19* metabolizer phenotype and *SLCO1B1* genotype on the sdLDL-C lowering effect of statins

For CYP2C19^*^2 (rs4244285, c.681G>A), the proportion of the GG, GA, and AA genotypes in patients with CAD were, respectively, 50.44, 41.59, and 7.96%, and the G and A allele frequencies were 71.24 and 28.76%, respectively (Table 5). For CYP2C19^*^3 (rs4986893, c.636G>A), the proportion of GG, GA, and AA were, respectively, 90.27, 9.73, and 0%, and the G and A allele frequencies were 95.13 and 4.87%, respectively (Table 5). The population with CYP1C19^*^2/^*^3 (GG/GG) was defined as extensive metabolizer (EM) phenotype, while the population with CYP2C19^*^2/^*^3 (GA/GG, GG/GA) and CYP1C19^*^2/^*^3 (GA/GA, AA/GG, GG/AA, AA/AA) were defined as intermediate metabolizer (IM) and poor metabolizer (PM) phenotype, respectively (24).

**Table 5:** Distribution of genotype and allelic frequency of polymorphisms of *SLCO1B1, CYP2C19* genes in CAD patients.

**SNPs**	**No. of patients (%)**				**Allele frequency**	
*SLCO1B1* *(rs4149056, c.521T>C)*	Total	TT	TC	CC	T	C
	172	137 (79.65%)	34 (19.77%)	1 (0.58%)	89.53%	10.47%
CYP2C19*2 *(rs4244285, c.681G>A)*	Total	GG	GA	AA	G	A
	113	57 (50.44%)	47 (41.59%)	9 (7.96%)	71.24%	28.76%
CYP2C19*3 *(rs4986893, c.636G>A)*	Total	GG	GA	AA	G	A
	113	102 (90.27%)	11 (9.73%)	0 (0%)	95.13%	4.87%

SNPs, single nucleotide polymorphisms.

**Figure 6:**
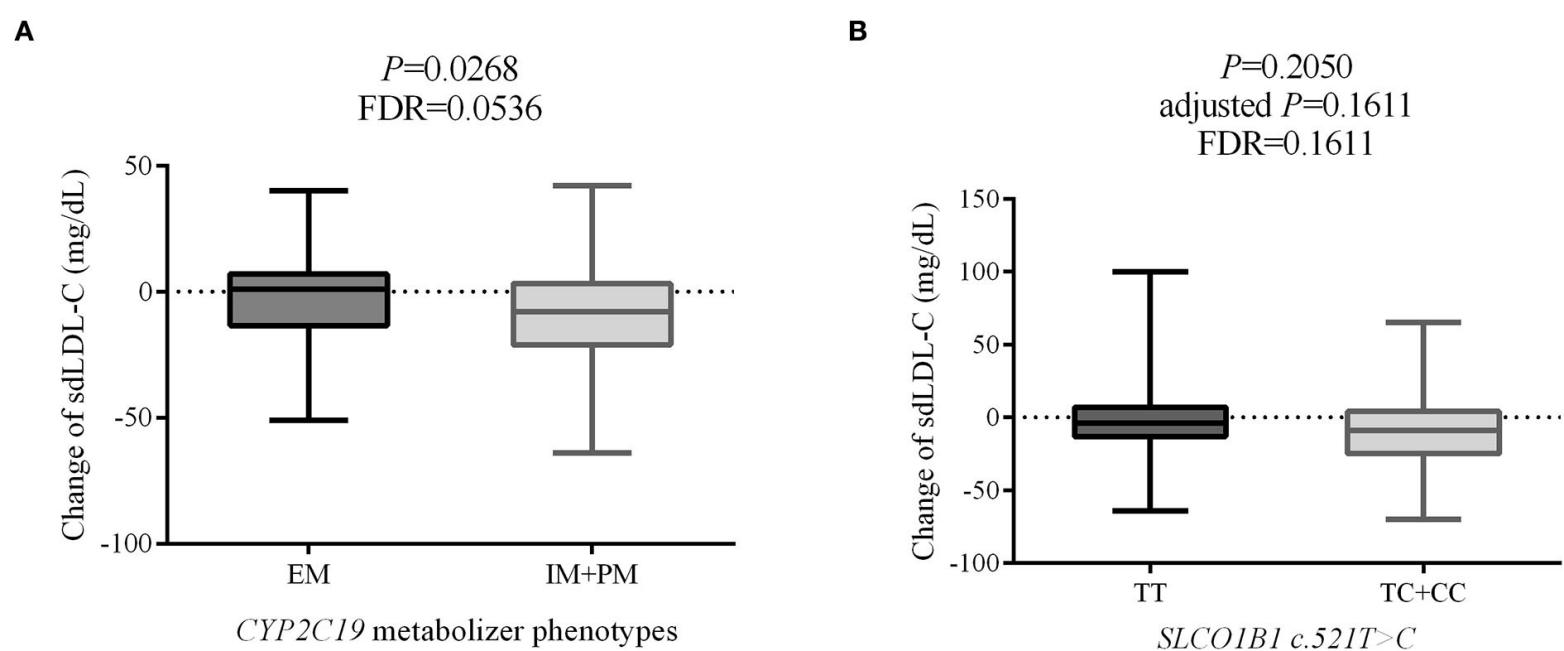
The effect on sdLDL-C lowering efficacy of statins of *CYP2C19* metabolizer phenotypes **(A)** and *SLCO1B1 c.521T*>*C* genotypes **(B)**. sdLDL-C, small dense low-density lipoprotein cholesterol; EM, extensive metabolizer; IM, intermediate metabolizer; PM, poor metabolizer; FDR, false discovery rate.

Change in individual sdLDL-C levels before and after statin treatment was calculated. Figure 6A indicates that patients with CAD with IM and PM *CYP2C19* metabolizer phenotype had more reduction (*P* = 0.0268, FDR = 0.0536) than in those patients with EM *CYP2C19* metabolizer phenotype. There was no significant difference in the distribution of clinical factors between the EM and IM+PM groups (Supplementary material 2). Table 6 shows that *CYP2C19* metabolizer phenotypes had no significant influence on any blood lipids in the patients with CAD before statin treatment. These results indicated that *CYP2C19* metabolizer phenotypes may affect the efficacy of statins in lowering sdLDL-C, and the patients with IM and PM phenotypes had better efficacy. Supplementary material 2 shows that there were significant differences in the distribution of gender (*P* = 0.045) and smoking history (*P* = 0.038) between the *SLCO1B1 c.521TT* and *c.521TC/TT* groups. After adjustment of covariances, the results indicated that the *SLCO1B* genotypes had no influence (adjusted *P* = 0.1611, FDR = 0.1611) on sdLDL-C lowering efficacy of statins (Figure 6B).

**Table 6:** Comparison of CAD patients' blood lipids according to *CYP2C19* metabolizer status.

**Blood lipids**	**EM** **(*n =* 49)**	**IM+PM** **(*n =* 64)**	***P*** **value**	**FDR**
TC	5.33 ± 1.25	5.08 ± 1.47	0.242	0.484
TG	1.43 ± 0.85	1.68 ± 1.20	0.211	0.492
HDL-C	1.13 ± 0.25	1.13 ± 0.57	0.175	0.613
LDL-C	3.55 ± 1.18	3.15 ± 1.26	0.076	1.000
LDLC-1	32.00 ± 15.26	27.61 ± 14.01	0.103	0.721
LDLC-2	33.78 ± 12.08	31.59 ± 15.13	0.187	0.524
LDLC-3	17.14 ± 9.28	18.03 ± 10.57	0.708	0.762
LDLC-4	7.80 ± 7.47	9.45 ± 8.53	0.369	0.646
LDLC-5	1.96 ± 2.44	2.81 ± 4.06	0.598	0.761
LDLC-6	0.33 ± 0.69	0.64 ± 3.29	0.504	0.706
LDLC-7	0.08 ± 0.40	0.70 ± 3.90	0.839	0.839
SdLDL-C	27.31 ± 18.07	31.64 ± 22.18	0.432	0.672
APO-A1	1.31 ± 0.22	1.29 ± 0.25	0.655	0.764
APOB	1.13 ± 0.34	1.04 ± 0.32	0.168	0.784

CAD, coronary artery disease; EM, extensive metabolizer; IM, intermediate metabolizer; PM, poor metabolizer; TC, total cholesterol; TG, triglyceride; HDL-C, high-density lipoprotein cholesterol; LDL-C, low-density lipoprotein cholesterol; sdLDL-C, small and dense low-density lipoprotein cholesterol; FDR, false discovery rate.

## 4. Discussion

Despite recent improvements in the treatment of lipid disorders and heart disease, ASCVD remains the major cause of death worldwide (25). Dyslipidemia is a well-known risk factor for ASCVD progression. In clinical practice, TC, TG, HDL-C, and LDL-C are the main markers for the primary and secondary prevention of ASCVD. However, for a substantial proportion of patients with ASCVD, the LDL-C level is in the normal range. It strongly limits the clinical significance of ASCVD screening and monitoring lipid-lowering efficacy. In recent years, increasing evidence suggests that sdLDL-C is a more effective biomarker for lipid disorder screening to prevent ASCVD (26–29).

In this study, 182 patients with CAD and 200 non-CAD controls were enrolled to validate the clinical value of sdLDL-C on CAD risk prediction. Although the LDL-C level was higher in patients with CAD than that in the control group, the ratio of CAD patients with elevated LDL-C was only 27.06%. A large proportion of patients with CAD could have been missed during health examinations because of normal LDL-C levels, which is consistent with previous studies and reports (11). For LDL-C subfractions, the LDL-C subfractions (LDLC-3 to LDLC-7) belonging to sdLDL-C were much higher in patients with CAD than those in controls. The ROC curve results showed that sdLDL-C (AUC = 0.838) and LDLC-4 (AUC = 0.835) have an advantage over LDL-C (AUC = 0.658) to screen patients with CAD from controls. Wu et al. reported that LDLC-4 played the most important role in CAD prediction by using machine learning models based on various factors (26). Chaudhary et al. showed that elevated LDLC-4 was associated with severe CAD (9). The current study accumulated evidence that LDLC-4 plays a crucial role in CAD risk screening. Therefore, LDL-C subfractions are necessary complements of total LDL-C for the primary prevention of CAD.

Statins are the mainstay of lipid-lowering therapy for patients with ASCVD. Clinically, LDL-C is the main target of lipid-lowering drugs to reduce ASCVD risk (4). The patients with heterozygous and homozygous carriers of the C allele at rs4149056 (^*^5) in *SLCO1B1* had a significantly increased risk for myopathy, compared with the patients with TT homozygotes, when taking statins (30). Meanwhile, clopidogrel is the most widely prescribed antiplatelet drug for secondary prevention of cardiovascular disease (31). As a prodrug, clopidogrel needs CYP450 enzymes to bio-convert it into corresponding active thiol metabolite (32). No-function variant allele of CYP2C19^*^2 and ^*^3 is common in the Chinese population, which leads to degraded or nonfunctional proteins (32). As a consequence, *SLCO1B1 c.521T*>C, CYP2C19^*^2, and ^*^3 have become combined companion diagnostics of a patient with CAD to guide their drug usage. However, only a few studies have investigated whether *SLCO1B1* genotypes and *CYP2C19* metabolizer phenotypes will influence sdLDL-C levels and the sdLDL-C lowering efficacy of statins.

Surprisingly, we found that *CYP2C19* metabolizer phenotypes had a significant impact on the therapeutic efficacy of statins. After statin treatment, the blood sdLDL-C level of the CAD patients with IM and PM *CYP2C19* metabolizer phenotypes decreased significantly more than that in patients with EM phenotype. *CYP450* is a group of isozymes that play a role in the phase 1 reactions of numerous exogenous drugs, such as statins. As a consequence, genetic polymorphisms in *CYP450* genes, such as *CYP3A4* and *CYP2C9*, can influence the metabolism of statins (33, 34). Bai et al. reported that *CYP2C19* genetic variations are associated with lipid metabolism in patients with ischemic stroke, and patients with ischemic stroke who were defined as poor *CYP2C19* metabolizers suffered a higher risk of palindromia (35). However, few researchers have reported the impact of *CYP2C19* metabolizer phenotypes on the efficacy of statins. Bailey et al. showed that genetic variations in *CYP2C19* cannot affect lipid-lowering efficacy (36). Finkelman et al. suggested that *CYP2C19* polymorphism does not affect rosuvastatin pharmacokinetics (37). To the best of our knowledge, this is the first study providing evidence that the *CYP2C19* metabolizer phenotypes may affect the efficacy of statins on sdLDL-C lowering. The possible mechanisms are as follows: The catalytic activity of cytochrome P450 proteins in drug metabolism was partially lost for patients with IM or PM *CYP2C19* metabolizer phenotypes. We speculate that the velocity of catalyzing statins to inactive metabolites is reduced in CAD patients with IM and PM *CYP2C19* metabolizer phenotypes. So, the half-life period of statins is prolonged. Notably, the power was 0.57 based on a sample size of 111 with a standard deviation of 18, which was calculated using the PASS 2021 software. The *P*-value was adjusted from 0.0268 to 0.0536 by multiple testing corrections. So, it needs further study with a large sample size of cohorts to validate the conclusion that whether *CYP2C19* metabolizer phenotypes affect the efficacy of statins in lowering sdLDL-C in patients with CAD.

*SLCO1B1* encodes a transporter that facilitates the hepatic uptake of statins (38). It is well-studied that genetic variation of *c.521T*>*C* in *SLCO1B1* is a genetic risk factor for statin myopathy (39). Sivkov et al. found that statin therapy was less effective in *SLCO1B1 c. 521CC* genotype carriers (40), who considered TC and LDL-C reduction as the efficacy of statins. However, a meta-analysis of 8 studies, including 2012 wild genotype (T/T) patients and 526 variant genotype (T/C and C/C) cases, found no significant association between the lipid-lowering efficacy of statins and the *SLCO1B1 c.521T*>*C* polymorphism (41). Another meta-analysis of Dai et al. also revealed no significant influence on the lipid-lowering efficacy of statins of *SLCO1B1* polymorphism (42). In this study, we also found that the *SLCO1B1* genotypes do not have a significant influence on sdLDL-C, lowering the efficacy of statins. Our study accumulated independent evidence to support the conclusion that no association can be found between the efficacy of statins and *SLCO1B1* polymorphism based on the sdLDL-C level.

## 5. Conclusion

In conclusion, LDLC-4 (AUC = 0.835) and sdLDL-C (AUC = 0.838) outperformed LDL-C (AUC = 0.658) in distinguishing patients with CAD from non-CAD controls. So, our study suggests that LDL-C subfractions are necessary supplements to traditional blood lipid detection for ASCVD primary prevention. In addition, we found the patients with IM and PM *CYP2C19* metabolizer phenotypes had better efficacy than patients with EM phenotypes. This study first provides evidence of *CYP2C19* metabolizer phenotypes, affecting the efficacy of statins on sdLDL-C lowering. SdLDL-C is an effective biomarker for both ASCVD risk screening and monitoring the efficacy of lipid-lowering therapy of statins.

## Data availability statement

The original contributions presented in the study are included in the article/Supplementary material, further inquiries can be directed to the corresponding author/s.

## Ethics statement

The studies involving human participants were reviewed and approved by the Ethics Committee of Quanzhou First Hospital Affiliated to Fujian Medical University and the First People's Hospital of Pingdingshan. The patients/participants provided their written informed consent to participate in this study.

## Author contributions

RD, TZ, and JZ designed the project. HZ, TZ, and WW collected samples and clinical data. JZ, ZH, YX, and XZ performed experiments and analyzed data. RD, JZ, XZ, and TZ wrote the manuscript. All authors read and approved the final manuscript.
