# Supplementary material for: CYP2C19 metabolizer phenotypes may affect the efficacy of statins on lowering small dense low-density lipoprotein cholesterol of patients with coronary artery disease
Source: Front Cardiovasc Med. 2022 Dec 19;9:1016126. doi: 10.3389/fcvm.2022.1016126 (PMC9806256; doi:10.3389/fcvm.2022.1016126)
Supplement: Supplementary file 1 [file Table_1.docx]

**Supplementary Table 1** The levels of blood lipids between controls and CAD patients by adjusting interactive effect of unbalanced clinical factors through general linear model of SPSS software.

| **Variables** | **Interactive effect between disease groups and clinical factors** | | | | | | **Controls** | **CAD patients** | **Adjusted *P* value** | **FDR** |
| --- | --- | --- | --- | --- | --- | --- | --- | --- | --- | --- |
|  | **Age** | **Gender** | **Hypertension** | **Diabetes** | **Smoke** | **Drink** | **（Mean±SD）** | **（Mean±SD）** |  |  |
| **TC (mmol/L)** | 0.004 | 0.402 | 0.4 | 0.911 | 0.043 | 0.004 | 4.333±0.205 | 5.338±0.122 | <0.001 | <0.001 |
| **TG (mmol/L)** | 0.003 | 0.021 | 0.916 | 0.41 | 0.312 | 0.224 | 1.415±0.096 | 1.533±0.071 | 0.013 | 0.014 |
| **HDL-C (mmol/L)** | 0.762 | 0.72 | 0.584 | 0.005 | 0.075 | 0.001 | 1.464±0.048 | 1.135±0.035 | <0.001 | <0.001 |
| **LDL-C (mmol/L)** | 0.085 | 0.159 | 0.531 | 0.39 | 0.105 | 0.128 | 2.566±0.077 | 3.312±0.084 | <0.001 | <0.001 |
| **SdLDL-C (mg/dL)** | <0.001 | 0.193 | 0.063 | 0.755 | 0.493 | 0.194 | 12.644±1.347 | 33.006±1.487 | <0.001 | <0.001 |
| **LDLC-1 (mg/dL)** | 0.859 | 0.087 | 0.027 | 0.864 | 0.01 | 0.11 | 29.198±2.147 | 29.476±1.220 | 0.911 | 0.911 |
| **LDLC-2 (mg/dL)** | 0.354 | 0.055 | 0.94 | 0.965 | 0.01 | 0.014 | 21.390±1.910 | 30.915±1.056 | <0.001 | <0.001 |
| **LDLC-3 (mg/dL)** | 0.004 | 0.249 | 0.068 | 0.775 | 0.482 | 0.071 | 9.499±0.730 | 18.449±0.806 | <0.001 | <0.001 |
| **LDLC-4 (mg/dL)** | <0.001 | 0.12 | 0.03 | 0.809 | 0.695 | 0.518 | 2.164±0.599 | 9.669±0.616 | <0.001 | <0.001 |
| **LDLC-5 (mg/dL)** | 0.022 | 0.815 | 0.279 | 0.883 | 0.742 | 0.928 | 0.403±0.235 | 3.174±0.259 | <0.001 | <0.001 |
| **LDLC-6 (mg/dL)** | 0.288 | 0.724 | 0.563 | 0.839 | 0.807 | 0.753 | 0.000±0.105 | 0.500±0.110 | 0.001 | 0.001 |
| **LDLC-7 (mg/dL)** | 0.042 | 0.417 | 0.41 | 0.84 | 0.443 | 0.641 | 0.000±0.125 | 0.568±0.138 | 0.002 | 0.003 |

Adjusted *P* value: *P* value by adjusting interactive effect between clinical factors and disease groups through general linear model.

FDR (False Discovery Rate): The Benjamini–Hochberg method was used to control the FDR.
