# Supplementary material for: CYP2C19 metabolizer phenotypes may affect the efficacy of statins on lowering small dense low-density lipoprotein cholesterol of patients with coronary artery disease
Source: Front Cardiovasc Med. 2022 Dec 19;9:1016126. doi: 10.3389/fcvm.2022.1016126 (PMC9806256; doi:10.3389/fcvm.2022.1016126)
Supplement: Supplementary file 2 [file Table_2.docx]

**Supplementary Table 2** Clinical characteristics of CAD patients according to *CYP2C19* metabolizer phenotypes and *SLCO1B1* SNPs.

|  | ***CYP2C19* metabolizer phenotypes** | | ***P* value** | ***SLCO1B1*** ***c.521T>C*** | | ***P* value** |
| --- | --- | --- | --- | --- | --- | --- |
|  | **EM**  **(n=49)** | **IM+PM (n=62)** |  | **TT**  **(n=130)** | **TC+CC**  **(n=31)** |  |
| **Age** | 63.29±12.08 | 63.90±12.04 | 0.566 | 64.49±11.83 | 63.77±12.60 | 0.921 |
| **Gender** |  |  | 0.310 |  |  | 0.045 |
| Male | 41 (83.7%) | 47 (75.8%) |  | 99 (76.2%) | 29 (93.5%) |  |
| Female | 8 (16.3%) | 15 (24.2%) |  | 31 (23.8%) | 2 (6.5%) |  |
| **BMI** | 23.69±3.03 | 23.54±2.83 | 0.609 | 23.99±3.05 | 24.35±2.82 | 0.600 |
| **Smoke** |  |  | 0.096 |  |  | 0.038 |
| Yes | 38 (77.6%) | 39 (62.9%) |  | 79 (60.8%) | 25 (80.6%) |  |
| No | 11 (22.4%) | 23 (37.1%) |  | 51 (39.2%) | 6 (19.4%) |  |
| **Drink** |  |  | 0.102 |  |  | 0.881 |
| Yes | 18 (71.2%) | 14(22.6%) |  | 82 (63.1%) | 20 (64.5%) |  |
| No | 31 (63.3%) | 48 (77.4%) |  | 48 (36.9%) | 11 (35.5%) |  |
| **Hypertension** |  |  | 0.265 |  |  | 0.881 |
| Yes | 29 (59.2%) | 43 (69.4%) |  | 82 (63.1%) | 20 (64.5%) |  |
| No | 20 (40.8%) | 19 (30.6%) |  | 48 (36.9%) | 11 (35.5%) |  |
| **Diabetes mellitus** |  |  | 0.265 |  |  | 0.643 |
| Yes | 17 (34.7%) | 28 (45.2%) |  | 52 (40.0%) | 11 (35.5%) |  |
| No | 32 (65.3%) | 34 (54.8%) |  | 78 (60.0%) | 20 (64.5%) |  |

EM: extensive metabolizer; IM: intermediate metabolizer; PM: poor metabolizer; BMI: Body Mass Index.
